# Occupational Happiness of Civilian Nurses in China: a cross-sectional study

**DOI:** 10.1186/s12912-023-01397-4

**Published:** 2023-07-04

**Authors:** Ying Meng, Xue Luo, Peng Sun, Yu Luo, Zonghua Wang, Lihua Wang, Yuhong Ge, Li Lin

**Affiliations:** 1 Department of Nursing Administration, School of Nursing, Army Medical University (Third Military Medical University), No.30 Gaotanyan Street, Shapingba District, Chongqing, China; 2 Medical Service Training Center,No.967 Hospital, Joint Logistics Support Force of PLA, NO.80 Shengli Road, Xigang District Dalian, China; 3 Department of Tropical Medicine, College of Military Preventive Medicine, Army Medical University(Third Military Medical University), No.30 Gaotanyan Street,Shapingba District Chongqing, China; 4 Section of Medical Education, Basic Medical College, Army Medical University (Third Military Medical University), No.30 Gaotanyan Street,Shapingba District Chongqing, China; 5 Department of Clinical Nursing, School of Nursing, Army Medical University(Third Military Medical University), No.30 Gaotanyan Street, Shapingba Distric Chongqing, China; 6 Admin Office of Southwest Hospital, Army Medical University (Third Military Medical University), No.29 Gaotanyan Street, Shapingba District Chongqing, China; 7 Department of General medicine, No.967 Hospital, Joint Logistics Support Force of PLA, NO.80 Shengli Road, Xigang District Dalian, China

**Keywords:** Military hospital, Civilian nurses, Occupational happiness, Questionnaire investigation, China

## Abstract

**Background:**

Civilian nurses have gradually become the main body of military nurses. Our study aimed to understand their occupational happiness and its influencing factors.

**Methods:**

This descriptive study was conducted with 319 civilian nurses working in 15 military hospitals in China. Based on literature review, expert consultation and combined with the characteristics of civilian positions, this study developed a questionnaire on occupational happiness of civilian nurses in military hospitals. The questionnaire includes 7 dimensions as follows: work emotion, salary, work environment, professional identity, work output, interpersonal relationship, well‑being. The demographic questionnaire and occupational well-being questionnaire of civilian nurses in military hospitals were analysed by *t-test*, analysis of variance and Pearson correlation analysis.

**Results:**

The occupational happiness score (3.83 ± 0.56, upper limit score: 5) was at the upper middle level. There were significant differences in occupational well-being by gender (*t* = -2.668, *p* = 0.008), age (*F* = 5.085, *p* = 0.007) and the type of city where the hospital was located (*F* = 15.959, *p* < 0.0001). The happiness score of females (3.94 ± 0.60) was higher than that of males (3.47 ± 0.54). Nurses who were over 41 years old had the highest occupational happiness. Compared with nurses younger than 30 years old, the p value was 0.004. The occupational happiness of nurses in hospitals in a “prefecture-level city” (*p* < 0.0001) and a “sub-provincial city” (*p* < 0.0001) was significantly higher than that of nurses in hospitals in a “municipality directly under the central government”. Correlation analysis showed that the higher the nurses’ satisfaction with professional identity, work output, work environment, salary, and interpersonal relationships, the higher their occupational happiness.

**Conclusion:**

Occupational happiness of civilian nurses in Chinese military hospitals was above the medium level. Gender, age, and the type of city where the hospital was located had a very significant impact on the level of occupational happiness. In addition, “professional identity”, “work output”, “work environment”, “salary”, and “interpersonal relationships” were significantly correlated with the occupational happiness of civilian nurses. They can be improved with some future lines of research.

## Background

Happiness is a subjective experience. Happiness mainly refers to directly experienced feelings of happiness, joy and pleasure as well as the evaluation of satisfaction with life, oneself and social relations based on the quality of life, which are among the essential characteristics of mental health [1–3]. Occupational happiness is a continuous subjective experience of happiness and a psychological state in which reasonable needs are met, potential is brought into play, and self-worth is realized by organizational members in a professional state. It can reflect an individual’s subjective feelings, such as job satisfaction and physical and mental pleasure [4, 5]. The higher occupational happiness is, the more individuals love their work [6].

In nursing work, high work pressure, widespread job burnout and high turnover intention seriously affect the quality of clinical nursing [7–9]. Improving occupational happiness can effectively reduce nurses’ job burnout, stimulate their subjective initiative to provide high-quality nursing services, reduce turnover intention, reduce the incidence of nursing errors and accidents, and ensure the life safety of patients [10, 11].

Civilian nurses are nonactive-duty nurses who are hired to work in military medical institutions and perform the same duties as active-duty officers in the same positions. While undertaking nursing work, they also undertake the task of medical support. Despite formal military training and standardized medical support training [12], military nurses have been gradually replaced by civilian nurses as the main nursing force in military hospitals [13]. The occupational happiness of Chinese civilian nurses has an important impact on the stability of the Chinese military nursing team and the improvement of nursing quality. In recent years, with the popularization and application of positive psychology in China, nurses’ occupational happiness has received increasing attention [14, 15]. However, existing studies focus on the occupational happiness of nurses in local hospitals, and there are few studies on the occupational happiness of civilian nurses in military hospitals [15–17]. Compared with nurses in local hospitals, civilian nurses in military hospitals may face a harsher and more complex natural environment and a high-intensity working environment [13]. What is the level of occupational happiness of civilian nurses in Chinese military hospitals? What factors do they face that affect their occupational happiness? Our study investigated civilian nurses in 15 military hospitals in China. This was a cross-sectional study conducted among 319 civilian nurses who worked in 15 military hospitals in China from June to July 2021. It aimed to understand their occupational happiness and its influencing factors.

## Materials and methods

### Participants

All civilian nurses in 15 hospitals constituted the study sample. We guaranteed all the participants the confidentiality of their private information and their right to withdraw from the study at any stage.

Inclusion criteria: ① informed consent, voluntary participation in this study; ② more than 1 year of employment.

The exclusion criteria were as follows: ① civilian nurses who were not in the unit for further study and training and ② civilian nurses working in nonclinical departments.

### Questionnaire

#### Demographic questionnaire

The questionnaire was designed based on a literature review and expert consultation. The contents of the questionnaire included gender, age, marital status, highest educational background, professional title, working years, and salary.

#### Occupational happiness questionnaire for civilian nurses in military hospitals

Based on a literature review, expert consultation and the characteristics of civilian positions, this study developed a questionnaire on the occupational happiness of civilian nurses in military hospitals, drawing on previous work such as the “Medical Workers” Occupational Happiness Scale [18] and the “Community Nurse Occupational Happiness Survey Questionnaire” [19]. The questionnaire included 7 dimensions (39 items): work emotion (3 items), salary (4 items), work environment (7 items), professional identity (8 items), work output (6 items), interpersonal relationships (8 items), and well‑being (3 items). All items were arranged randomly. A five-grade Likert evaluation method (very high inconformity: 1; some inconformity: 2; general conformity: 3; some conformity: 4; very high conformity: 5) was used to grade the psychological evaluation of individuals. Among these items, 8 negative topics were included. The higher the score, the higher the occupational happiness. Fifty civilian nurses were randomly selected for pre-investigation. We conducted a correlation analysis between the 39 items on the pre-survey questionnaires and the total score of happiness. The double-tailed significance values for all 39 items were found to be less than 0.05. Additionally, the Cronbach Alpha value of the scale (Cronbach) was 0.916, indicating a high homogeneity between the survey items and the overall scale, and a high reliability of the scale. Using Amos software for confirmatory factor analysis (CFA), the loading of each item was higher than 0.5 only in a single dimension, which suggests valid items and passes the validity test.

#### Methods for data collection

For data collection, this study utilized the online survey platform “Questionnaire Star”. The questionnaire was imported into the platform and set up to only be answered through WeChat. Additionally, each registered WeChat account was only allowed to complete one questionnaire. The questionnaire was designed to require answers for each question, and the system automatically identified missing items and reminded survey subjects before submission. The research team selected 15 military hospitals and distributed a questionnaire via a QR code to the WeChat group of civilian nurses in each hospital. The purpose and instructions for filling out the survey were explained, with an emphasis on the anonymity and confidentiality of the responses. Participation in the survey was voluntary. The research team carefully reviewed each questionnaire after they were collected. A total of 319 questionnaires were collected in this survey, and the effective rate was 100%.

### Statistical analysis

SPSS 22.0 software was used for statistical analysis. The measurement data are described by mean and standard deviation. The t-test is used to analyse group differences in continuous variables, whereas one-way analysis of variance (ANOVA) is employed to test for differences in other factors, with the exception of gender. Pearson’s correlation analysis, on the other hand, is utilized to examine the correlation between various dimensions. The difference was statistically significant with a two-tailed probability value of < 0.05 or 0.01.

## Results

### Characteristics of the study sample

A total of 319 civilian nurses from military hospitals participated in the study (Table 1). There were 12 males (3.76%) and 307 females (96.24%), which is in line with the composition ratio of male and female nursing professionals in China. There were 233 nurses aged 31–40 (73%) and 258 married nurses (80.9%). A total of 285 nurses had bachelor’s degrees (89.3%), 166 nurses had the title of chief nurse (52.0%), and the working years of 246 nurses were more than 10 years (77.1%). A total of 207 nurses (64.9%) had a monthly salary of more than 10,000 yuan (RMB).

**Table 1:** The characteristics of the study participants (n = 319)

Type		Number(%)
Gender	Male	12(3.8)
	Female	307(96.2)
Age	18–30	50(15.7)
	31–40	233(73.0)
	>41	36(11.3)
Marital status	Single	50(15.7)
	Married	258(80.9)
	Divorce	11(3.4)
Highest educational background	College	26(8.2)
	Bachelor	285(89.3)
	Master	8(2.5)
Professional title	Nurse	12(3.8)
	Nurse practitioner	136(42.6)
	Nurse in charge	166(52.0)
	Deputy chief nurse	4(1.3)
	Chief nurse	1(0.3)
Working years	1–5	27(8.5)
	6–10	46(14.4)
	>10	246(77.1)
Salary (RMB)	5000–10,000 yuan	112(35.1)
	>10,000 yuan	207(64.9)
Type of city where the hospital is located	Municipality directly under the central government	113(35.4)
	Sub-provincial city	66(20.7)
	Prefecture-level city	134(42.0)
	County-level city	6(1.9)

### Current situation analysis of occupational happiness

**Fig. 1:**
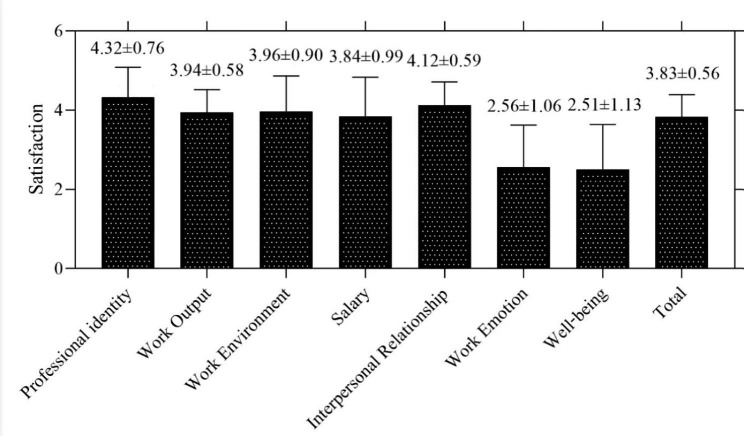
Comparison of various dimensions of occupational happiness of civilian nurses

The average occupational happiness score of the respondents was 3.83 ± 0.56. Among the seven dimensions, the scores for professional identity, work output, work environment, salary, and interpersonal relationships were close to or higher than 4 (Fig. 1). The average score for work emotion and well-being was less than 3 (Fig. 1). In this study, the occupational happiness of civilian nurses was generally at the upper middle level. They were highly satisfied with the five dimensions of professional identity, work output, working environment, salary, and interpersonal relationships. However, they had a lack of confidence in work emotion and well-being.

### Variance analysis of occupational happiness

#### Gender

There were significant differences in occupational well-being by gender (*t*=-2.668, *p* = 0.008, Table 2). The happiness score of females (3.94 ± 0.60, Table 2) was higher than that of males (3.47 ± 0.54, Table 2). Among the seven dimensions, there were four dimensions with significant gender differences: professional identity (*t*=-2.637, *p* = 0.009, Table 2), work output (*t*=-2.469, *p* = 0.014, Table 2), salary (*t*=-2.644, *p* = 0.009, Table 2), and interpersonal relationships (*t*=-3.187, *p* = 0.002, Table 2). In these four dimensions, female satisfaction was higher than male satisfaction.

**Table 2:** Comparison of gender differences in occupational happiness of civilian nurses

	Male	Female	*t*	*p*
Professional identity	3.75 ± 0.80	4.34 ± 0.76	-2.637	0.009**
Work output	3.75 ± 0.70	4.28 ± 0.73	-2.469	0.014*
Work environment	3.55 ± 1.03	3.97 ± 0.89	-1.615	0.107
Salary	3.10 ± 0.84	3.87 ± 0.99	-2.644	0.009**
Interpersonal relationships	3.75 ± 0.71	4.38 ± 0.67	-3.187	0.002**
Work emotion	2.42 ± 1.12	2.20 ± 1.13	0.655	0.513
Well‑being	2.64 ± 0.94	2.51 ± 1.14	0.400	0.689
Total	3.47 ± 0.54	3.94 ± 0.60	-2.668	0.008**

*Correlation is significant at the 0.05 level (2-tailed)

** Correlation is significant at the 0.01 level (2-tailed)

### Age

According to Table 3, the comparison of the average occupational happiness of civilian nurses by age (F = 5.085, *p* = 0.007) revealed significant differences in all seven dimensions. Nurses who were over 41 years old had the highest occupational happiness. Compared with nurses younger than 30 years old, the p value was 0.004. Compared with nurses aged 31–40, the p value was 0.003. There was no difference in happiness between nurses aged 18–30 and nurses aged 31–40. The results suggest that the occupational happiness of young civilian nurses needs to be improved.

**Table 3:** Comparison of age differences in occupational happiness of civilian nurses

	Age			F	*p*
	<30	31–40	>41		
Professional identity	4.14 ± 0.74	4.29 ± 0.78	4.75 ± 0.46	7.759	0.001**
Work output	4.11 ± 0.74	4.23 ± 0.75	4.68 ± 0.43	7.363	0.001**
Work environment	3.85 ± 0.85	3.92 ± 0.92	4.32 ± 0.75	3.500	0.031*
Salary	3.63 ± 0.97	3.8 ± 1.01	4.41 ± 0.7	7.678	0.001**
Interpersonal relationships	4.2 ± 0.69	4.34 ± 0.7	4.72 ± 0.36	6.763	0.001**
Work emotion	2.73 ± 1.2	2.16 ± 1.09	1.81 ± 1.02	8.230	0.000***
Well‑being	2.79 ± 1.2	2.53 ± 1.1	1.99 ± 1.07	5.516	0.004**
Total	3.84 ± 0.65	3.9 ± 0.61	4.22 ± 0.36	5.085	0.007**

*Correlation is significant at the 0.05 level (2-tailed)

** Correlation is significant at the 0.01 level (2-tailed)

### Marital status

There was no significant difference in terms of marital status (F = 0.643, *p* = 0.526, Table 4). Notably, marital status led to a significant difference in “work emotion” (*p* = 0.015). The occupational happiness scores for single nurses were higher than others. The results of multiple comparisons showed that there was a significant difference in “work emotion” between “single” and “married” nurses (*p* = 0.004).

**Table 4:** Comparison of marital status differences in the occupational happiness of civilian nurses

	Single	Married	Divorce	*F*	*p*
Professional identity	4.16 ± 0.79	4.35 ± 0.75	4.32 ± 0.82	1.317	0.270
Work output	4.12 ± 0.81	4.28 ± 0.72	4.44 ± 0.77	1.256	0.286
Work environment	3.89 ± 0.9	3.95 ± 0.9	4.34 ± 0.59	1.143	0.320
Salary	3.61 ± 1.02	3.87 ± 0.99	4.16 ± 0.74	2.114	0.122
Interpersonal relationships	4.21 ± 0.77	4.39 ± 0.67	4.4 ± 0.68	1.429	0.241
Work emotion	2.63 ± 1.2	2.13 ± 1.09	2.09 ± 1.36	4.255	0.015*
Well‑being	2.85 ± 1.16	2.44 ± 1.11	2.52 ± 1.23	2.783	0.063
Total	3.86 ± 0.7	3.93 ± 0.59	4.06 ± 0.54	0.643	0.526

*Correlation is significant at the 0.05 level (2-tailed)

### Highest educational background

There was no significant difference in highest educational background (F = 0.374, *p* = 0.688, Table 5). However, for “work emotion” (*p* = 0.015) and “well‑being” (*p* = 0.012), educational differences led to a significant difference. The results of multiple comparisons showed that the correlation of “work emotion” and “well‑being” with “master’s degree” were higher than “college” (*p* = 0.005; *p* = 0.003) and “bachelor’s” (*p* = 0.005; *p* = 0.005).

**Table 5:** Comparison of the highest educational background differences in the occupational happiness of civilian nurses

	College	Bachelor	Master	*F*	*p*
Professional identity	4.44 ± 0.61	4.31 ± 0.78	4.03 ± 0.72	0.887	0.413
Work output	4.47 ± 0.6	4.25 ± 0.74	3.98 ± 0.73	1.700	0.184
Work environment	4.04 ± 0.73	3.96 ± 0.91	3.71 ± 0.9	0.400	0.671
Salary	3.92 ± 0.97	3.85 ± 0.98	3.19 ± 1.41	1.851	0.159
Interpersonal relationships	4.46 ± 0.61	4.36 ± 0.69	4.05 ± 0.67	1.093	0.337
Work emotion	2.04 ± 1.14	2.19 ± 1.11	3.31 ± 1.22	4.237	0.015*
Well‑being	2.29 ± 1.14	2.5 ± 1.12	3.63 ± 0.88	4.490	0.012*
Total	4 ± 0.45	3.92 ± 0.61	3.8 ± 0.79	0.374	0.688

*Correlation is significant at the 0.05 level (2-tailed)

** Correlation is significant at the 0.01 level (2-tailed)

### Professional title

There was no significant difference in professional title (F = 0.592, *p* = 0.669). However, it is important to note that the chief nurse had the highest score for occupational happiness, but this result should be interpreted with caution as only one person participated in the survey and therefore, is not representative. Overall, the study suggests that the level of occupational happiness among nurses and nurse practitioners is relatively high. After analysing the scores across seven dimensions, it was found that nurses gave the highest rating to “work effectiveness” and “work environment”. On the other hand, nurse practitioners rated “profession itself”, “salary”, and “interpersonal relationships” the highest. The nurse in charge gave the same rating as the nurse practitioner for “interpersonal relationships”, while the deputy chief nurse rated “work emotions” and “physical health” the highest.

### Working years

There was no significant difference in working years (F = 2.147, *p* = 0.119, Table 6). However, the difference in working years led to significant differences in “professional identity” (*p* = 0.020), “salary” (*p* = 0.040), “interpersonal relationships” (*p* = 0.011) and “work emotion” (*p* = 0.001). The results of multiple comparisons suggested that the occupational happiness in “professional identity” of nurses with more than 10 working years was higher than that of nurses with 6–10 working years (*p* = 0.017). Nurses who had worked for more than 10 years had higher occupational happiness in “salary” than nurses who had worked for 1–5 years (*p* = 0.015). Nurses who had worked for more than 10 years had higher occupational happiness in “interpersonal relationships” than nurses who had worked for 6–10 years (*p* = 0.007). Nurses who had worked for 6–10 years had higher occupational happiness in “work emotion” than nurses who had worked for more than 10 years (*p* < 0.001).

**Table 6:** Comparison of working years differences in the occupational happiness of civilian nurses

	1–5 years	6–10 years	> 10 years	*F*	*p*
Professional identity	4.11 ± 0.78	4.09 ± 0.75	4.38 ± 0.76	3.984	0.020*
Work output	4.11 ± 0.79	4.07 ± 0.7	4.31 ± 0.73	2.641	0.073
Work environment	3.8 ± 0.89	3.77 ± 0.87	4.01 ± 0.9	1.751	0.175
Salary	3.42 ± 1.05	3.74 ± 0.87	3.9 ± 1	3.258	0.040*
Interpersonal relationships	4.19 ± 0.7	4.13 ± 0.74	4.42 ± 0.66	4.611	0.011*
Work emotion	2.46 ± 1.22	2.72 ± 1.15	2.08 ± 1.09	7.135	0.001**
Well‑being	2.67 ± 1.19	2.8 ± 1.17	2.44 ± 1.11	2.333	0.099
Total	3.78 ± 0.67	3.81 ± 0.63	3.96 ± 0.59	2.147	0.119

*Correlation is significant at the 0.05 level (2-tailed)

** Correlation is significant at the 0.01 level (2-tailed)

### Salary

There was no significant difference in salary (F = 0.289, *p* = 0.773). However, it is worth noting that the “50-10000” salary group scored relatively high on dimensions such as “profession itself”, “work effectiveness”, “work environment”, “work emotion”, and “physical health”; “>10000” group scores high on salary and interpersonal relationships dimensions.

### Type of city where the hospital is located

There was a significant difference in the type of city where the hospital was located (F = 15.959, *p* < 0.0001, Table 7). In the total score of occupational happiness, “prefecture-level cities” (*p* < 0.0001) and “sub-provincial cities” (*p* < 0.0001) were significantly higher than “municipalities directly under the central government”. “sub-provincial cities” (*p* < 0.0001), “prefecture-level cities” (*p* < 0.0001) and “county-level cities” (*p* = 0.039) had significantly higher occupational happiness than “municipalities directly under the central government” in “professional identity”. In “professional identity”, the occupational happiness of “prefecture-level cities” (*p* = 0.038) was significantly higher than that of “sub-provincial cities”. In “work output”, the occupational happiness of “county-level cities” (*p* = 0.046), “prefecture-level cities” (*p* < 0.0001) and “sub-provincial cities” (*p* < 0.0001) was significantly higher than that of “municipalities directly under the central government”. For “working environment”, the occupational happiness of “prefecture-level cities” (*p* < 0.0001) and “sub-provincial cities” (*p* = 0.006) was significantly higher than that of “municipalities directly under the central government”. The occupational happiness of “county-level cities” (*p* = 0.007), “prefecture-level cities” (*p* < 0.0001) and “sub-provincial cities” (*p* < 0.0001) was significantly higher than that of “municipalities directly under the central government” in “salary”. “Prefecture-level cities” (*p* = 0.008) had significantly higher occupational happiness than “sub-provincial cities” in “salary”. The occupational happiness of “prefecture-level cities” (*p* < 0.0001) and “sub-provincial cities” (*p* < 0.0001) was significantly higher than that of “municipalities directly under the central government” in “interpersonal relationships”. The occupational happiness of “municipalities directly under the central government” was significantly higher than that of “county-level cities” (*p* = 0.025), “prefecture-level cities” (*p* < 0.0001) and “sub-provincial cities” (*p* < 0.0001) in “work emotion”. The occupational happiness of “municipalities directly under the central government” was significantly higher than that of “county-level cities” (*p* = 0.009), “prefecture-level cities” (*p* < 0.0001) and “sub-provincial cities” (*p* < 0.0001) in “well‑being”.

**Table 7:** Comparison of differences in the type of city where the hospital is in the occupational happiness of civilian nurses

	Municipality directly under the central government	Subprovincial city	Prefecture-level city	County-level city	*F*	*p*
Professional identity	3.89 ± 0.85	4.4 ± 0.66	4.62 ± 0.56	4.5 ± 0.48	22.738	< 0.0001***
Work output	3.87 ± 0.78	4.38 ± 0.64	4.53 ± 0.6	4.43 ± 0.51	20.421	< 0.0001***
Work environment	3.64 ± 0.91	4.01 ± 0.84	4.19 ± 0.85	4.1 ± 0.53	8.548	< 0.0001***
Salary	3.16 ± 1.05	3.98 ± 0.79	4.33 ± 0.67	4.13 ± 0.52	39.977	< 0.0001***
Interpersonal relationships	4.05 ± 0.74	4.46 ± 0.64	4.57 ± 0.56	4.5 ± 0.63	14.110	< 0.0001***
Work emotion	2.62 ± 1.04	1.99 ± 1.13	2 ± 1.12	1.58 ± 0.92	8.434	< 0.0001***
Well‑being	2.96 ± 1	2.36 ± 1.16	2.24 ± 1.11	1.78 ± 0.86	10.769	< 0.0001***
Total	3.64 ± 0.69	3.98 ± 0.53	4.14 ± 0.47	3.98 ± 0.29	15.959	< 0.0001***

*Correlation is significant at the 0.05 level (2-tailed)

** Correlation is significant at the 0.01 level (2-tailed)

### Correlation analysis between various dimensions and overall occupational happiness of civilian nurses

Pearson correlation analysis was used to explore the correlation between the scores of “professional identity”, “work output”, “work environment”, “salary”, “interpersonal relationships”, “work emotion”, and “well-being” and the total score of occupational happiness (Table 8). There was no significant correlation between “work emotion”, “well-being” and the total score of occupational happiness (*p* > 0.05). There was a significant positive correlation between “professional identity”, “work output”, “work environment”, “salary”, and “interpersonal relationships” and the total score of occupational happiness (*p* < 0.05). “Professional identity” had the highest correlation with the total score of occupational happiness (correlation coefficient 0.936), followed by “work output” (correlation coefficient 0.918). In short, the higher the satisfaction with “professional identity”, “work output”, “work environment”, “salary”, and “interpersonal relationships”, the higher the occupational happiness of civilian nurses.

**Table 8:** Correlation analysis between various dimensions and the occupational happiness of civilian nurses

	Professional identity	Work output	Work environment	Salary	Interpersonal Relationship	Work Emotion	Well‑being	Total
Professional identity	1							
Work output	0.919**	1						
Work environment	0.849**	0.821**	1					
Salary	0.812**	0.777**	0.773**	1				
Interpersonal relationships	0.872**	0.878**	0.797**	0.696**	1			
Work emotion	− 0.325**	-0.301**	-0.281**	-0.212**	-0.311**	1		
Well‑being	− 0.306**	-0.296**	-0.291**	-0.267**	-0.314**	0.790**	1	
Total	0.936**	0.918**	0.905**	0.851**	0.886**	-0.103	-0.100	1

*Correlation is significant at the 0.05 level (2-tailed)

** Correlation is significant at the 0.01 level (2-tailed)

## Discussion

In this study, the total score of occupational happiness of civilian nurses in Chinese military hospitals (3.83 ± 0.56) was above the medium level. Other research reports from China show that the overall occupational happiness of military hospital nurses (including soldiers, civilians, and non-staff) and local hospital nurses (including staff and non-staff) is at a medium level [6, 20, 21]. The reason may be that military civilians have advantages in welfare, career choice, career stability and career development and more superiority among peers. The object of this study is civilian nurses in military hospitals, who are military personnel and have higher job stability and more career development opportunities. According to Chinese career policy, the salary and welfare benefits of civilian nurses are higher than those of nurses in local hospitals [22]. They cannot be dismissed by the hospital without gross negligence, but they still have the right to apply for resignation or terminate the employment contract independently [23]. Therefore, civilian nurses are more stable than non-staff nurses and have more career choices than military nurses. In addition, civilian nurses enjoy some of the same priority treatment as active-duty military nurses, which makes them feel honoured. The above factors make civilian nurses more satisfied with their “professional identity”, “work output”, “work environment”, “salary”, and “interpersonal relationships”. Because most civilian nurses did not graduate from military medical colleges, they have not received systematic military physical training and lack professional training in military medicine. However, they participate in military training and non-war military medical support tasks according as needed [12]. There is a gap for active-duty military nurses in physical fitness and military skills, so physical health is an issue of great pressure and challenges. Their feelings about work in military hospitals were in the lower middle level.

In this study, there were significant differences in the occupational happiness of civilian nurses of different genders. Women scored higher than men. Influenced by traditional ideas, women still dominate the nursing industry in China, and male nurses tend to have a low sense of professional identity and personal value [24]. When patients meet male nurses, they often have different degrees of prejudice and discrimination, which causes conflicts between nurses and patients [25]. This increases the psychological pressure of male nurses and reduces their occupational happiness. In this study, the older the civilian nurses were, the longer their working years were and the higher their occupational happiness was. The occupational happiness of civilian nurses over 41 years old was higher than that of civilian nurses of other ages in all dimensions. Civilian nurses who had worked for more than 10 years were more satisfied with “interpersonal relationships” and “work emotions”. The reason might be that with the increase in age and working years, the abilities of civilian nurses in all aspects were improved, which made them easier to recognize at work; furthermore, the salary was better, so their occupational happiness increased. We suggest that male nurses and civilian nurses under the age of 41 should pay more attention to their work and life. They should be encouraged to engage with the real environment, constantly improve their practical ability, communication ability and management ability, win the respect of patients with high-quality service, feel professional value, and improve their career development potential. At the same time, nursing managers can use job rotation, psychological counselling, and other ways to help nurses reduce pressure and improve their occupational happiness.

In this study, the type of city where the hospital was located was the main factor affecting the occupational happiness of civilian nurses. The occupational happiness of civilian nurses located in “prefecture-level cities” and “sub-provincial cities” was higher than that of civilian nurses in “municipalities directly under the central government”. The “prefecture level city”, “sub provincial city”, and “municipalities directly under the central government” are the administrative levels of Chinese cities. The administrative level of " municipalities directly under the central government” is higher than that of " sub-provincial cities “. The administrative level of “sub-provincial cities” is higher than that of “prefecture-level cities”. The administrative level of cities determines their different socio-economic management authorities, leading to differences in economic development levels. Therefore, the economic development level of “sub provincial cities” is higher than that of “prefecture level cities”. The reason might be that the economic development and consumption level of “prefecture-level cities” and “sub-provincial cities” are moderate. If civilian nurses can reasonably distribute their salaries, they can not only ensure a higher quality of life but also realize wealth accumulation. Therefore, their occupational happiness is naturally relatively high. However, the economic development of “municipalities directly under the central government” is relatively fast, and the consumption level is relatively high. Living in “municipalities directly under the central government” with the same wage income would produce economic pressure in education and housing, resulting in differences in the quality of life of civilian nurses [23]. Studies have shown a positive correlation between occupational happiness and quality of life, so a decline in quality of life would also be accompanied by a decrease in satisfaction with professional identity, working environment, salary, and other aspects [4, 26–28]. However, it is worth noting that the satisfaction of civilian nurses working in municipalities was significantly higher than that of civilian nurses in the other three regions in “work emotions” and “well-being”. This might be because the four municipalities directly under the central government in China are basically superior to other types of cities regarding education, science and technology, economy, culture, and transportation and are the places where most people want to live. Civilian nurses cherish job opportunities in the military hospitals of municipalities directly under the central government and have strong positive work emotion, which promotes a virtuous cycle of physical and mental health. Therefore, the nursing managers of military hospitals in municipalities directly under the central government should pay more attention to the family and living conditions of civilian nurses, encourage them to talk about difficulties they encounter and try to help them solve their problems. At the same time, it is necessary to provide more training opportunities for civilian nurses to broaden their horizons and improve their abilities. Nurses can improve their occupational happiness by improving their sense of occupational benefits.

Based on Pearson correlation analysis, we found that “professional identity”, “work output”, “work environment”, “salary”, and “interpersonal relationships” were significantly positively correlated with occupational happiness. However, occupational happiness was not related to “work emotion” and “well-being”. The reason might be that occupational happiness mainly comes from professional identity [29]. When nurses’ efforts are recognized by patients, family members, colleagues and leaders, their occupational happiness improves, in the opposite situation, occupational happiness decreases. Civilian nurses have a solid theoretical foundation, high practical skills, and high work enthusiasm. Once they are actively involved in work, it is easy to achieve results and obtain recognition. The recognition of the people around them can encourage them to work harder. This cycle can greatly stimulate their enthusiasm and interest in work and help them to obtain a higher sense of happiness in their career. The regulations on civilian personnel by the Chinese people’s Liberation Army clearly stipulate that civilian personnel enjoy the same status and wages as active servicepeople. When nurses believe they are treated fairly, they have more trust in organizations and managers and work with hope and motivation. Equal status and stable income make civilian nurses feel recognized by the hospital, so they are highly satisfied with the professional environment.

### Strengths and limitations

The Strengths of this study include the sample covers a wide area and the gender characteristics were in line with the actual characteristics of Chinese nursing professionals. 319 civilian nurses were from 15 military hospitals in China, which were in different types of cities. At the same time, their sex composition ratio conforms to the gender characteristics of Chinese nursing professionals. This research design could understand the overall situation of professional well-being of Chinese civilian nurses.

There are also some limitations in this study. The validation of the questionnaire is not complete so the data can be biased. Other identity type nurses working in the same unit, such as soldiers and non-staff nurses, were not selected for the control study. Further in-depth studies were recommended in future studies.

## Conclusion

In general, the occupational happiness of civilian nurses in Chinese military hospitals was above the medium level. Gender, age, and the type of city where the hospital was located had a very significant impact on the level of occupational happiness. In addition, “professional identity”, “work output”, “work environment”, “salary”, and “interpersonal relationships” were significantly correlated with the occupational happiness of civilian nurses. They can be improved with some future lines of research.

## Data Availability

The datasets used and analysed during the current study were available from the corresponding author on reasonable request.
